# Biomechanical Behavior of Composite Bone–Osteosynthesis Constructs in Complex Proximal Humerus Fractures: A Synergistic Experimental and Finite Element Approach

**DOI:** 10.3390/bioengineering13060625

**Published:** 2026-05-27

**Authors:** Andrei Scripcaru, Vasile Iulian Antoniac, Mădălina Maria Diac, Mihnea Theodor Sîrbu, Tatiana Iov, Veronica Scripcaru, Simona Irina Damian, Diana Bulgaru Iliescu, Norin Forna, Paul-Dan Sîrbu

**Affiliations:** 1 Faculty of Medicine, Grigore T. Popa University of Medicine and Pharmacy Iasi, 700115 Iasi, Romania; andrei.c.scripcaru@umfiasi.ro (A.S.); madalina-maria.diac@umfiasi.ro (M.M.D.); mihnea-theodor.sirbu@umfiasi.ro (M.T.S.); veronica.scripcaru@umfiasi.ro (V.S.); simona.damian@umfiasi.ro (S.I.D.); diana.bulgaru@umfiasi.ro (D.B.I.); norin.forna@umfiasi.ro (N.F.); paul.sirbu@umfiasi.ro (P.-D.S.); 2 Institute of Legal Medicine, 700455 Iasi, Romania; 3 Faculty of Materials Science and Engineering, National University of Science and Technology Politehnica Bucharest, 313 Splaiul Independentei Street, 060042 Bucharest, Romania; antoniac.iulian@gmail.com; 4 Faculty of Medicine and Pharmacy, “Dunărea de Jos” University, 800008 Galati, Romania; 5 Faculty of Medicine, Apollonia University, 700511 Iasi, Romania

**Keywords:** proximal humerus fracture, osteosynthesis, locking plates, intramedullary nail, biomechanical testing, finite element analysis, composite bone, mechanical stiffness

## Abstract

This study evaluates the mechanical behavior of bone-implant assemblies used in treating complex proximal humerus fractures, a clinical challenge due to the anisotropic nature of bone and variability in patient-specific conditions. The aim of this study was to compare the stability and stress distribution of three fixation methods: polyaxial locking plates, monoaxial locking plates, and intramedullary nails. Using 4th-generation composite humerus models, a four-part fracture (Neer IV) was simulated. The assemblies underwent axial compression testing using a universal testing machine, complemented by finite element analysis (FEA) and stereomicroscopy. The results indicate that while both plate types exhibited similar mechanical behavior—with stiffness values around 113–115 N/mm and failure initiated by plastic deformation of the implant—the intramedullary nail configuration demonstrated higher stiffness values under the tested experimental conditions (1084 N/mm), approximately 9.5 times higher than that of the plates. However, the nail assembly failed through brittle fracture of the bone rather than implant deformation. We conclude that while the intramedullary nail configuration demonstrated higher stiffness under the tested experimental conditions, its performance is heavily dependent on bone quality. In contrast, locking plates may provide a more gradual load-transfer behavior by transferring a greater proportion of the mechanical load to the implant, potentially making them more suitable for osteoporotic bone conditions, where reducing excessive stress concentration within the bone tissue may be beneficial.

## 1. Introduction

The mechanical assessment of assemblies comprising heterogeneous materials remains a significant challenge in biomechanical engineering. Evaluating the interaction between human bone—an anisotropic, living tissue with properties that fluctuate based on age and systemic health—and metallic osteosynthesis implants requires a precise understanding of stress transfer and deformation limits [1]. While Finite Element Analysis (FEA) serves as a valuable tool for estimating these behaviors, its predictive accuracy is reliable primarily within the elastic range. FEA has become an important instrument in orthopedic biomechanics, being increasingly used for the investigation of stress redistribution, strain concentration and load-transfer mechanisms in pathological bone structures. Previous computational studies have highlighted the relevance of finite element modeling in the biomechanical assessment of osteoarthritic and fractured bone, particularly through the integration of experimentally derived loading conditions, anatomical constraints, muscular forces and contact interactions [2]. Such approaches can contribute to the identification of mechanically vulnerable regions and support the optimization of fixation strategies and implant design. Once stresses exceed the material’s elastic limit, particularly in the transition to plastic deformation, the predictive accuracy of computational models may decrease significantly, necessitating empirical validation through physical testing [3,4].

Proximal humerus fractures account for approximately 5–6% of all adult fractures [5,6], especially among the elderly population where osteoporosis is a prevalent complicating factor [7]. The surgical management of these complex fractures, particularly four-part fractures according to the Neer classification, remains a subject of ongoing debate in the orthopedic community [8,9]. Current stabilization strategies primarily involve locked osteosynthesis plates or intramedullary nails. However, evidence and clinical opinions diverge regarding which method offers the optimal balance between mechanical stability and biological preservation.

Some researchers argue that locked plates with angular stability, whether monoaxial or polyaxial, provide superior fixation by creating a fixed-angle construct that resists varus collapse, a common failure mode in osteoporotic bone [10]. Conversely, others advocate for intramedullary nails, suggesting that their centromedullary position allows for better load sharing and requires less soft tissue disruption, potentially leading to faster biological healing [11,12]. Despite these advancements, clinical complications such as screw perforation, hardware failure, and loss of reduction persist [13,14], highlighting the need for a deeper understanding of the assembly’s mechanical limits.

The purpose of this study is to evaluate and compare the mechanical behavior of the bone-implant assembly in complex proximal humerus fractures using three distinct fixation methods: a polyaxial locked plate (A1), a monoaxial locked plate (A2), and an intramedullary nail (A3). By utilizing 4th-generation composite humerus models, which provide a standardized alternative to cadaveric bone, we aimed to eliminate the variables associated with biological diversity and focus strictly on the mechanical performance of the constructs by identifying the critical stress zones and failure modes of each assembly under axial compression. Axial compression was selected as the sole loading condition because it provides a controlled and reproducible baseline scenario for comparing construct stiffness, stress transfer, and early failure mechanisms under standardized experimental conditions. However, this loading protocol should be interpreted primarily as a simplified screening model rather than a fully representative simulation of the complex multidirectional loading conditions encountered in vivo at the shoulder joint.

## 2. Materials and Methods

To ensure the reproducibility of the study and eliminate the variables associated with the biological diversity of human tissue, the experimental assemblies were constructed using standardized artificial humerus models.

### 2.1. Bone Models and Fracture Simulation

The study utilized a fourth-generation composite humerus produced by Sawbones^®^ (model #3404, Pacific Research Laboratories, Vashon, WA, USA). According to the manufacturer’s technical specifications, the model has the following dimensions: length (a) = 365 mm, head diameter (b) = 55 mm, neck diameter (c) = 23 mm, and proximal width (d) = 64 mm. The intramedullary canal is pre-reamed from 5.5 mm to 80 mm starting from the distal end.

The material properties of this composite bone include a density of 0.27 g/cm^3^, a compressive modulus of elasticity of 155 MPa, and a compressive strength of 6 MPa.

To simulate a clinical scenario, a complex four-part proximal fracture was created according to the Neer classification (Neer IV), involving the surgical neck and the greater and lesser tuberosities.

### 2.2. Osteosynthesis Implants

Three types of fixation assemblies were prepared using the following implants:

- Assembly A1: A locked plate with polyaxial angular stability (INTERCUS GmbH, Rudolstadt, Germany) made of titanium alloy with variable angle holes allowing multidirectional screw placement;
- Assembly A2: A locked plate with monoaxial angular stability (INTERCUS GmbH, Rudolstadt, Germany) made of titanium alloy with fixed angle holes;
- Assembly A3: An intramedullary nail (TRIGEN Proximal Humerus Nail, Smith & Nephew, Memphis, TN, USA), 80 mm length, 8–7 mm diameter, trapezoidal proximal section with ~25° multidirectional locking screw angles for stable fixation.

For the plates, proximal locking screws with lengths of 48 mm and 50 mm were used to ensure optimal fragment fixation. The geometry of both plates was similar, differing primarily in the reaming angle of the fixation holes. For the intramedullary nail, fixation was achieved using four 44 mm cancellous proximal locking screws and two 26 mm cortical distal locking screws.

### 2.3. Specimen Preparation and Fixation

**Figure 1:**
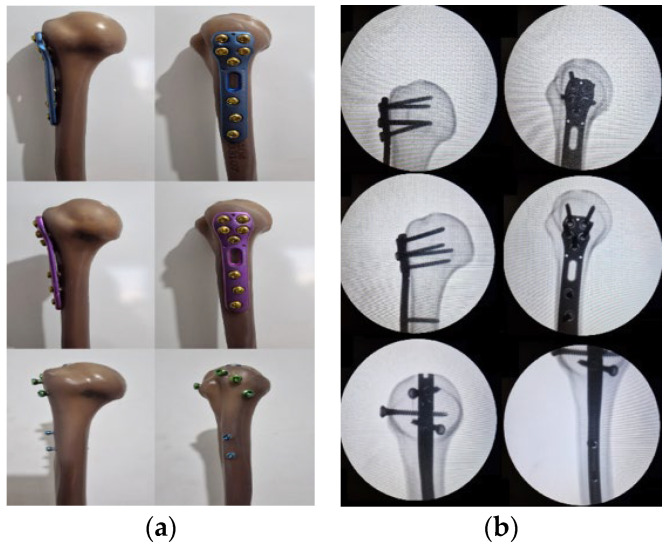
(**a**)—Initial mounting of the implants on the intact composite bone; (**b**) Radiological verification of implant and screw positioning.

**Figure 2:**
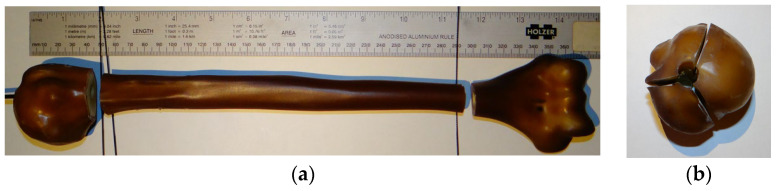
(**a**) Surgical neck and distal epiphysis osteotomy; (**b**) Osteotomies for the simulation of a Neer IV fracture.

**Figure 3:**
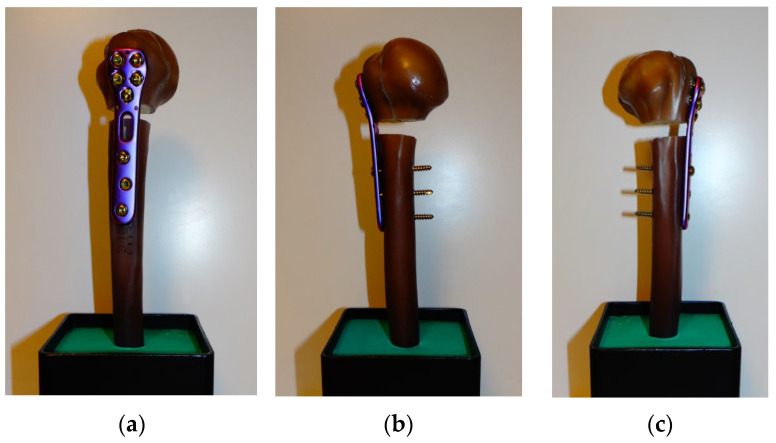

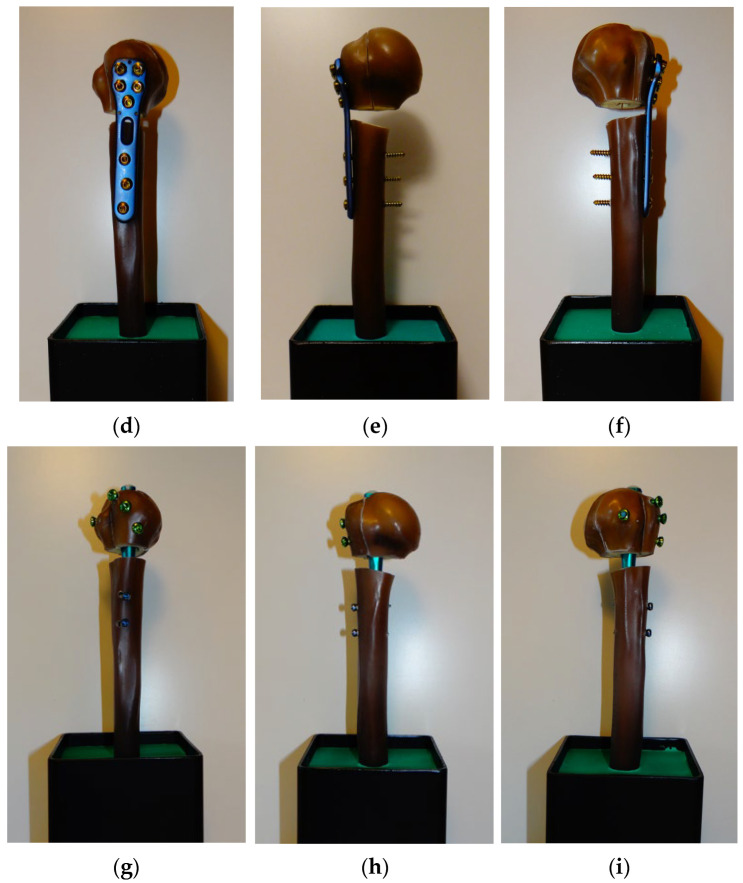
Final assembly aspects. (**a**–**c**) Lateral, posterior and anterior views of the composite bone–polyaxial locking plate assembly; (**d**–**f**) lateral, posterior and anterior views of the composite bone–monoaxial locking plate assembly; (**g**–**i**) lateral, posterior and anterior views of the composite bone–intramedullary nail assembly.

The preparation followed a strict protocol:

- Initial mounting of the implants on the intact composite bone to ensure correct alignment, verified via conventional radiography (Figure 1);
- Removal of the implants to perform the fracture osteotomies and removal of the distal epiphysis (Figure 2);
- Remounting of the implants using the pre-formed holes;
- The distal ends of the assemblies were secured in 10/10/10 cm metal supports using SHERASOCKEL–FLUSSIG dental cement (SHERA Werkstoff-Technologie GmbH & Co. KG, Germania, Lemförde, Germany) to provide a stable base for mechanical testing (Figure 3). The use of SHERASOCKEL–FLUSSIG dental cement was preferred over PMMA or other embedding materials due to its high compressive strength (≈50 MPa), elevated surface hardness (≈230–250 N/mm^2^), and low setting expansion (≈0.05%), which ensure superior dimensional stability, reproducible fixation conditions, and minimization of artefacts during biomechanical testing.

For simplification, the assemblies were coded based on the type of implant used: the assembly with polyaxial plate was coded A1, the one with monoaxial plate was coded A2, and the assembly using the intramedullary nail was coded A3.

### 2.4. Mechanical Testing Protocol

These assemblies were mechanically tested in compression at the Faculty of Materials Science and Engineering of the Polytechnic University of Bucharest using a Walter + Bai LFW 300 universal testing machine (Walter + Bai AG, Löhningen, Switzerland), utilizing a system adapted for non-standardized testing.

**Figure 4:**
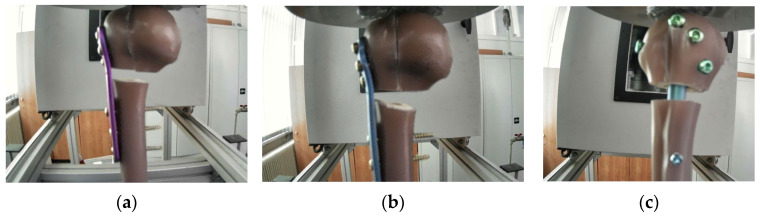
Initiation of axial compression mechanical testing for the artificial bone/osteosynthesis material assemblies using the Walter + Bai LFW 300 universal testing machine. (**a**) polyaxial angular stability locked plate; (**b**) monoaxial angular stability locked plate; (**c**) intramedullary nail.

The assemblies rested on a flat surface made of hardened tool steel with a hardness of 65HRC to avoid system deformations. The load was applied axially in compression, with the application point being the area of maximum curvature of the humeral head (Figure 4).

The adopted loading protocol was intended as a simplified and reproducible comparative biomechanical screening model and did not aim to fully reproduce the complex multidirectional loading conditions encountered in vivo at the shoulder joint.

Test parameters were as follows: the trial was displacement-controlled at a speed of 5 mm/min to avoid varying stress rates. The end criterion for the test was either the failure of an assembly component or considerable plastic deformations produced during testing.

Since the testing was non-standardized, the experimental results were recorded using the testing machine’s dedicated software, DionPRO V5.2 (Walter + Bai AG, Switzerland), and subsequently processed using Mathematica software, Version 13.0 (Wolfram Research Inc., Champaign, IL, USA) to determine a series of parameters that allow for a quantitative comparison of the mechanical behavior.

### 2.5. Computational and Microscopic Analysis

To visualize the stress distribution within the implant, a Finite Element Analysis (FEA) was performed using a simplified model of the system, removing secondary components and applying the load directly to the implant in a manner that describes the deformation observed during the experiment. The simulation was conducted using the analysis tools available in the SolidWorks Simulation environment (version 2021, Dassault Systèmes, Dassault Systèmes SolidWorks Corp., Waltham, MA, USA).

Given the geometric similarity of the plates, the model used for the simulation maintained the same configuration and dimensions, with the only difference being the fixation holes, which were positioned at different angles.

**Figure 5:**
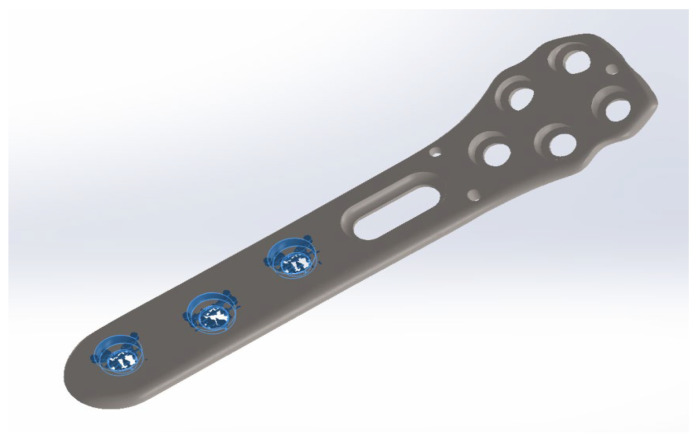
Application mode of the fixation zones locations.

In the configuration of experimental assemblies A1 and A2 (Figure 3a–f), it can be observed that the fixed zones can be considered as the three holes at the level of the humeral diaphysis; consequently, the model is simplified based on this consideration, with fixation constraints being applied to this region (Figure 5).

**Figure 6:**
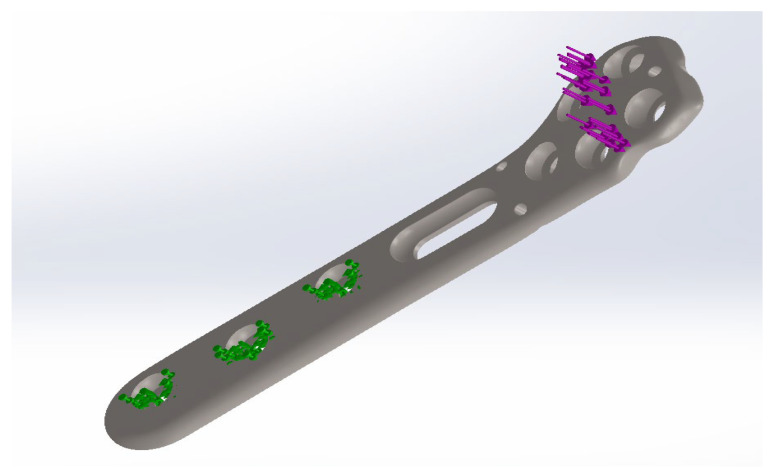
Application of force-type loads (pink colored vectors) and fixed constraints applied (indicated in green colored vectors).

Axial compression loading will generate a mixed stress state in the median region of the plate; therefore, the applied load was a force oriented perpendicularly to the plate, in such a way as to apply a moment (Figure 6). The fixation constraints were applied to the diaphyseal fixation holes, according to the simplified model described above.

Since the material is less relevant under the specific conditions of this analysis, it was parameterized as commercially pure titanium, with a modulus of elasticity of 110 GPa, a Poisson’s ratio of 0.3, a tensile strength of 235 MPa, and a yield strength of 140 MPa.

After the discretization of the model, using the meshing tools available in SolidWorks Simulation, the simulation was performed, and the model was analyzed in comparison with the experimental assembly.

A similar protocol was used for the intramedullary nail, simplifying the entire assembly to a load applied strictly to the nail. Accordingly, based on experimental observations, the holes securing the nail in the diaphyseal region were considered as fixed geometry.

**Figure 7:**
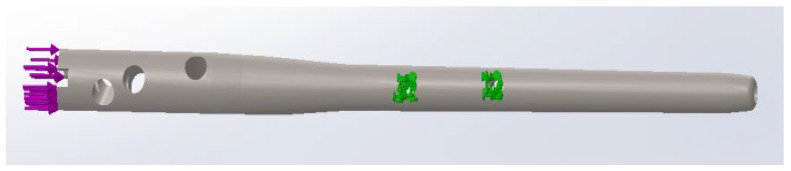
Application of a force-type load on the nail (indicated in pink colored vectors) and fixed constraints applied (indicated in green colored vectors).

In this context, due to the high stiffness of the assembly, an axial application of the force-type load was employed (Figure 7).

The material was defined as commercially pure titanium, using the same parameters described above for the locked plates.

The present simulations did not include the complete bone–implant–screw construct or contact interactions between bone, screws, and implant components. Therefore, the FEA results were interpreted qualitatively and used only to support the experimental observations regarding stress concentration and deformation patterns.

Following the discretization of the component, the simulation was performed and the Von Mises stress distribution, principal strains, and displacement fields were extracted and qualitatively compared with the experimentally observed deformation patterns.

As the finite element simulations were performed using the predefined analysis tools available in SolidWorks Simulation, no custom mathematical formulation was developed in the present study. The numerical evaluation was based on the standard finite element workflow implemented in the software, using the material parameters specified above and the boundary conditions defined according to the simplified experimental configuration.

After the mechanical tests, the assessment of highly stressed or failed areas within the assemblies was conducted through macroscopic analysis using a Swift SM101 stereomicroscope (Swift Optical Instruments, Inc. (Motic Swift Line), Universal City, TX, USA), equipped with a video camera for image acquisition.

## 3. Results

After processing the data obtained during the mechanical testing of the assemblies, two parameters were determined for comparative analysis: assembly stiffness and the yield point, defined as the point at which the force–displacement relationship becomes non-linear.

Associated with the experimental measurements, the results of the macroscopic/stereomicroscopic fractographic analysis performed on the assembly components and the finite element analysis results are also presented.

### 3.1. Assembly A1

**Figure 8:**
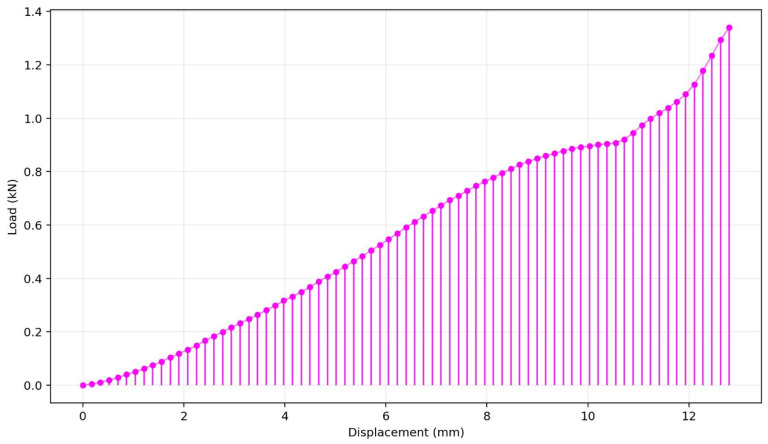
Force–displacement curve obtained during the testing (A1).

Following the testing of assembly A1, which utilizes the polyaxial angular stability locked plate, a displacement–force curve was obtained (Figure 8).

**Figure 9:**
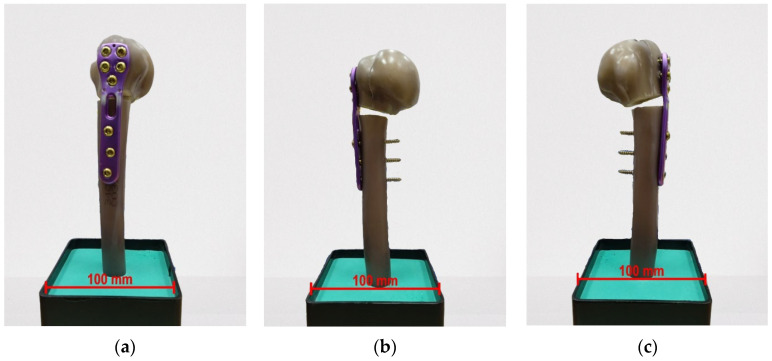
Appearance of the assembly after testing, highlighting the plastic deformations in the proximal fixation and the free regions of the plate. (**a**) lateral view; (**b**) posterior view; (**c**) anterior view.

The behavior of the assembly indicates a linear force variation at the beginning of the test, followed by an inflection that transitions into a short plateau, subsequently followed by a sharp increase in force. This suggests the initiation of plastic deformation within the plate (the transition to the plateau region). The sudden increase in force is caused by the contact between the composite bone components, which illustrate the displacement of the assembly components. During the test, the proximal and medial components of the humerus were in contact, subsequently separating slightly due to elastic recovery (Figure 9).

The processing of the experimental curve allowed for an estimated stiffness of 113 N/mm and a proportionality limit between force and displacement of 773 N.

**Figure 10:**
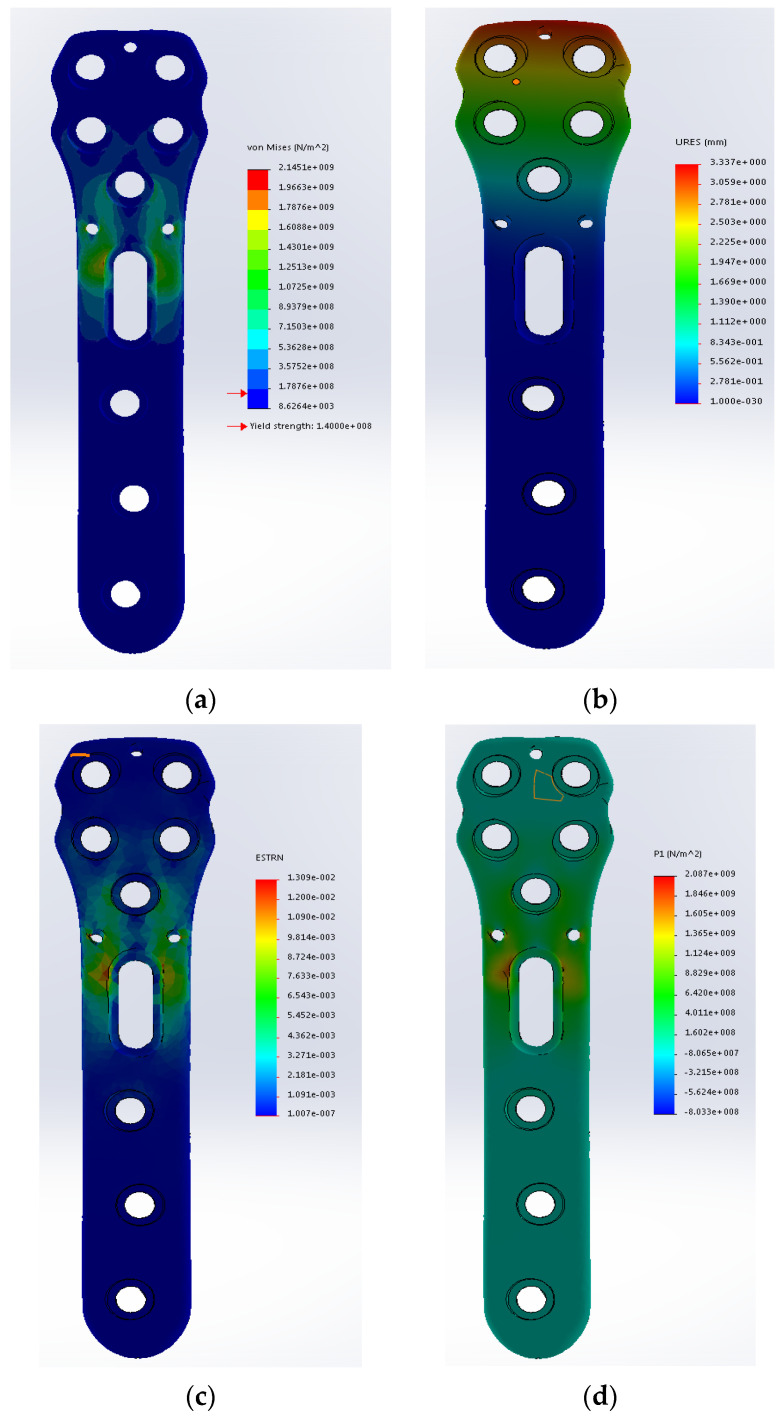
Results of the static finite element analysis performed on the polyaxial plate. (**a**) Von Mises equivalent stresses; (**b**) Displacements; (**c**) Equivalent von Mises strains; (**d**) Principal stresses along the Ox direction.

Using the mechanical behavior alongside the plastically deformed regions, the simplified model for the finite element analysis described above was parameterized (Figure 10).

**Figure 11:**
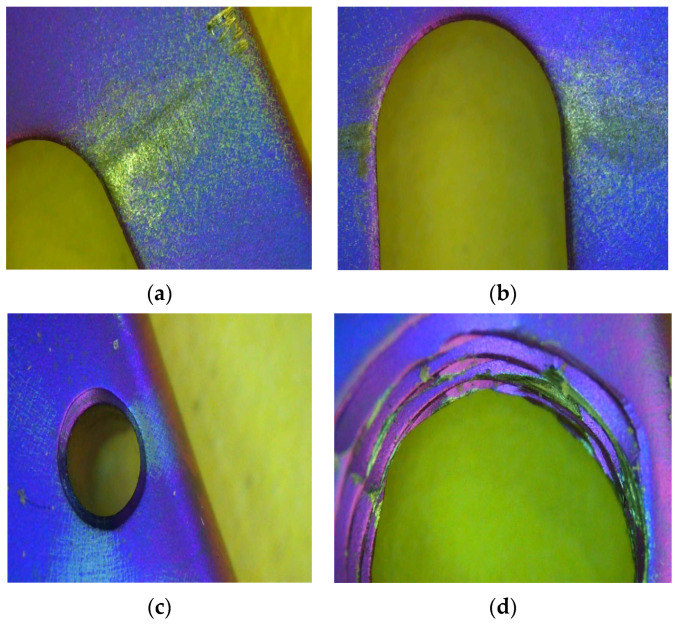
Aspects of the deformed regions on the A1 assembly: (**a**) macroscopic view of the deformation at the level of the surgical neck; (**b**) secondary aspect of the deformed region near the surgical neck; (**c**) detailed view of an affected zone at the surgical neck level; (**d**) microscopic aspect of thread/hole deformation.

The simulation results correspond with the displacements (Figure 10b) observed during the experiment, and the equivalent strains (Figure 10c) exhibit a pattern similar to those observed in Figure 9a and Figure 11a–c.

These results, associated with the fractographic analysis performed using the stereomicroscope in critical regions, allow for an assessment of the stress distribution during mechanical loading.

**Figure 12:**
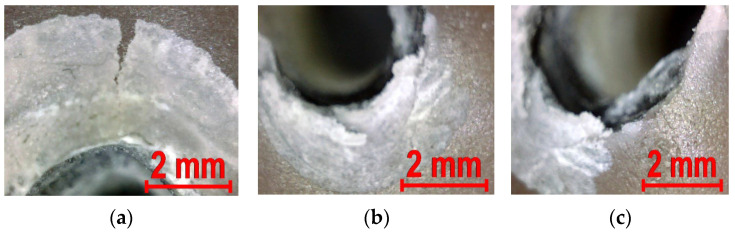
Plastic deformations observed in the proximal region screw holes of assembly A1: (**a**) view of screw holes plastic deformation; (**b**) secondary view of screw holes plastic deformation; (**c**) detailed view of the plastic deformation.

In the case of assembly A1, the highest amplitude stress is found in the free region of the plate, within the bone defect area at the level of the surgical neck; its value exceeds the yield strength of the material. The stress transfer to the plate is achieved through the fixation screws in the proximal region of the assembly; in that specific bone area, deformations are observed at the level of the screw holes (Figure 12).

In the configuration of assembly A1, the polyaxial plate bears the majority of the stresses; the axial load is converted into a bending stress due to the assembly’s configuration, and plastic deformation initiates at 723 N.

### 3.2. Assembly A2

**Figure 13:**
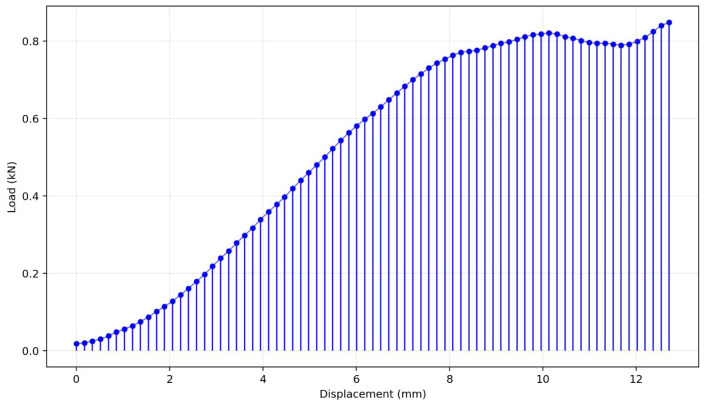
Force–displacement curve obtained during the testing (A2).

Assembly A2, configured using a monoaxial plate for fixation, was tested under similar conditions to assembly A1. The assembly’s behavior is characterized by an initial linear variation, followed by a plateau region indicating the plastic deformation of the plate, and finally, a sharp increase in force (Figure 13).

**Figure 14:**
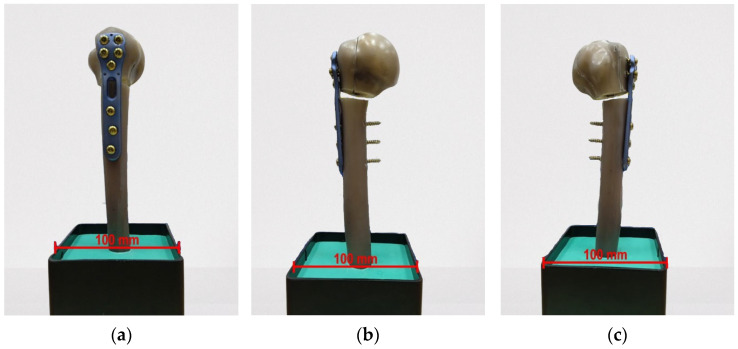
Appearance of assembly A2 after testing. (**a**) lateral view; (**b**) posterior view; (**c**) anterior view.

The presence of this plateau on the force–displacement curve suggests the initiation of plastic deformation, a phenomenon visually confirmed in Figure 14a by visible surface discoloration and deformation patterns in the free section of the plate (the bone defect area at the level of the surgical neck). This sudden force increase is triggered when the proximal and medial fragments of the artificial bone come into direct contact. From a mechanical standpoint, the plate is subjected to bending stress, as evidenced by the state of the assembly at the end of the test, shown in Figure 14b,c. Testing was maintained until the two bone components established contact, at which point the slope of the curve rises abruptly. Following the processing of the experimental data, a stiffness of 115 N/mm and a proportionality limit of 610 N were determined.

To conduct a more detailed investigation of the stresses and strains, finite element analysis (FEA) was employed, using the same simplified model previously described. The plate geometry was adjusted accordingly to match the physical counterpart used in the experimental tests.

**Figure 15:**
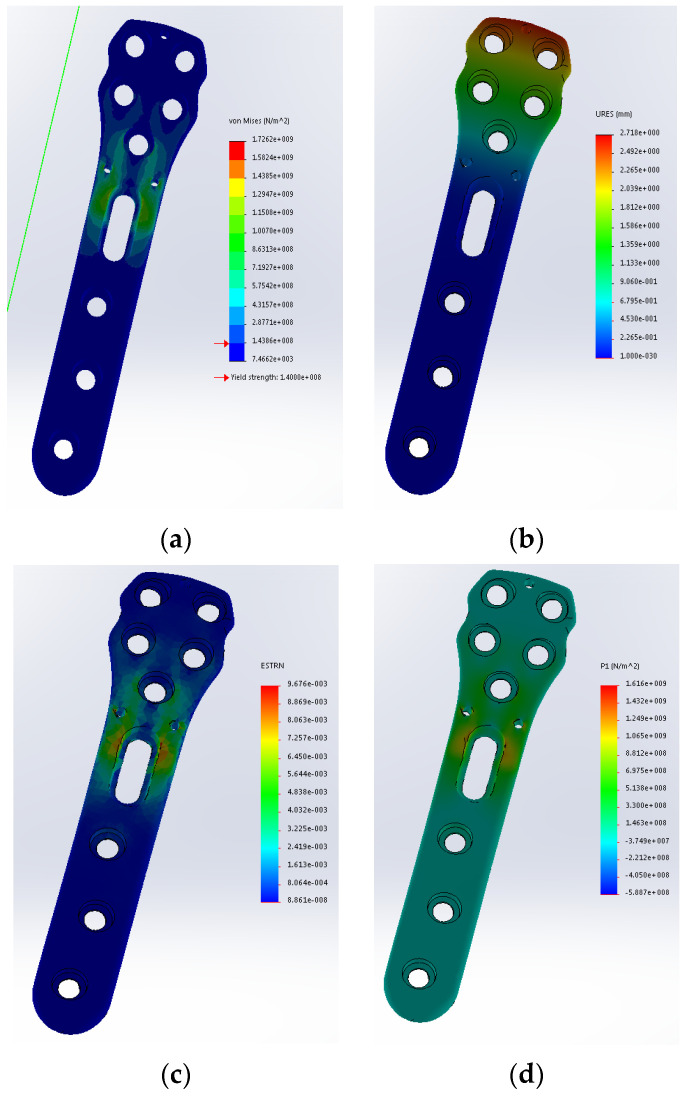
Results of the static finite element analysis performed on the monoaxial plate. (**a**) Von Mises equivalent stresses; (**b**) Displacements; (**c**) Equivalent von Mises strains; (**d**) Principal stresses along the Ox direction.

The displacements indicated by the finite element analysis (Figure 15b) are in full agreement with the behavior observed during the experimental testing of the assembly, with the proximal component recording the highest displacement.

**Figure 16:**
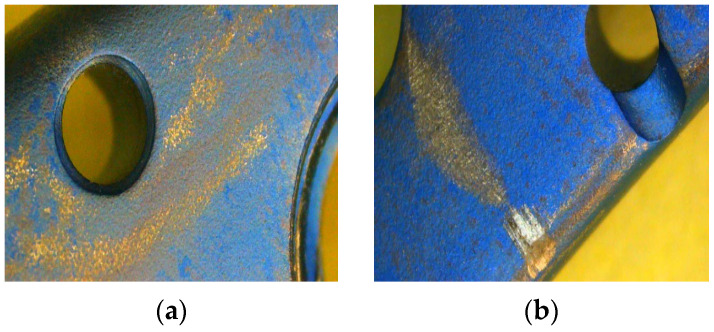
Detailed views of the deformed regions on the surface of the A2 assembly plate. (**a**) Screw holes in the proximal region; (**b**) Area of the plate corresponding with the surgical neck.

Furthermore, the stress and strain distribution within the monoaxial plate (Figure 15a,c,d) matches the pattern identified through the macroscopic analysis presented in Figure 14a and Figure 16a,b.

The correlation between the stress and strain distribution predicted by finite element analysis and the stereomicroscopic observations allows for an evaluation of the load transmission stages within assembly A2. The load is initially taken up by the proximal component of the artificial humerus and transmitted via the screws to the plate. The plate bears the majority of the stresses, showing tensioned regions that lead to plastic deformation through bending (Figure 16a,b). In contrast, the diaphyseal fixation area of the plate is subjected to low-amplitude stresses.

**Figure 17:**
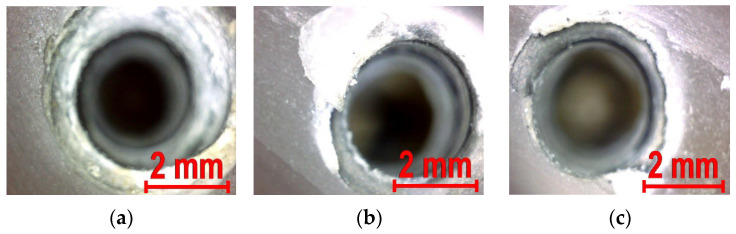
Plastic deformations observed in the proximal region screw holes of assembly A2: (**a**) view of screw holes plastic deformation; (**b**) secondary view of screw holes plastic deformation; (**c**) detailed view of the plastic deformation.

The composite material of the artificial bone, in the proximal region, shows signs of more intense loading, manifested through material deformation (Figure 17).

### 3.3. Assembly A3

**Figure 18:**
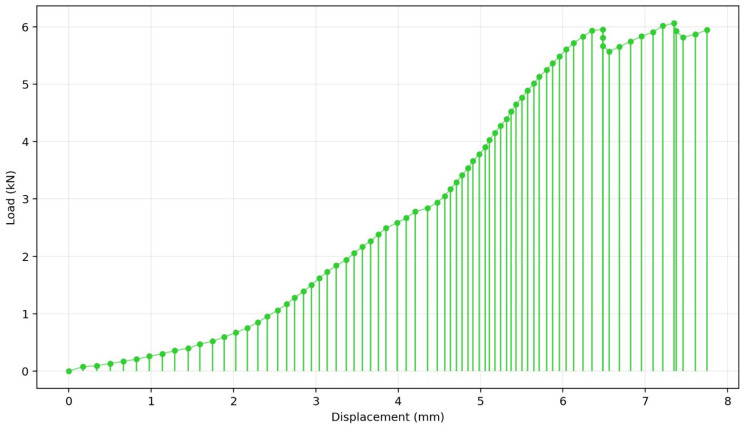
Force–displacement curve obtained during experimental testing (A3).

Assembly A3 was configured using an intramedullary nail as the osteosynthesis device. The force–displacement curve (Figure 18) reveals three distinct regions: the first corresponds to the stabilization of the assembly through compression and the reduction in gaps between components; the second represents the effective load transfer to the assembly components; and the transition between the second and third regions, marked by a discontinuity in the curve, suggests the failure of an assembly component. In this instance, no regions attributable to plastic deformation were observed, as failure occurred in a brittle manner and was associated with the fracture of the artificial bone. By processing the curve, an assembly stiffness of 1084 N/mm and a proportionality limit of 2514 N were estimated.

**Figure 19:**
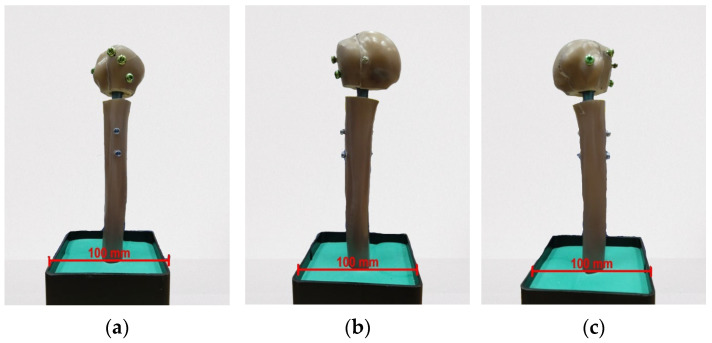
Appearance of assembly A3 after testing. (**a**) lateral view; (**b**) posterior view; (**c**) anterior view.

The axial compressive loading did not produce any evident deformations in the assembly (Figure 19).

**Figure 20:**
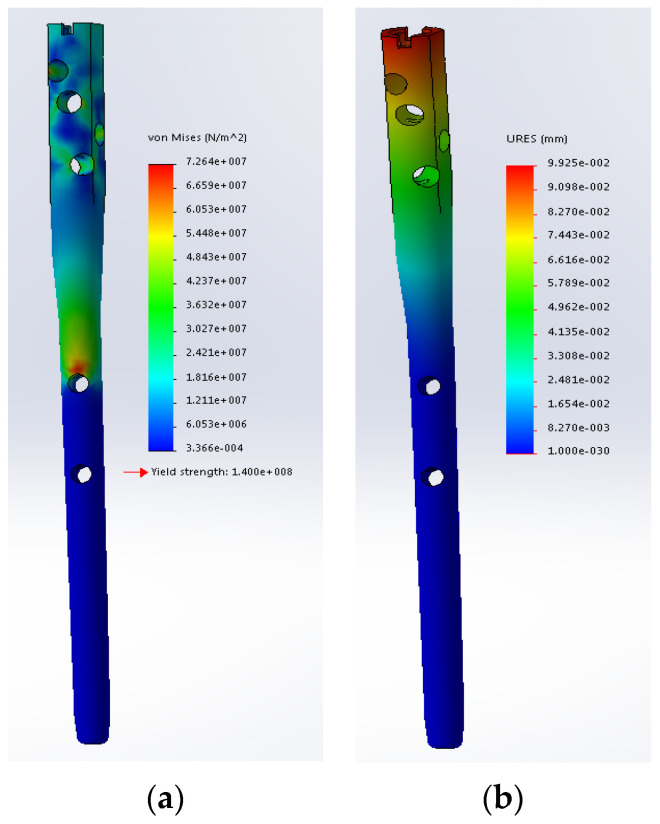

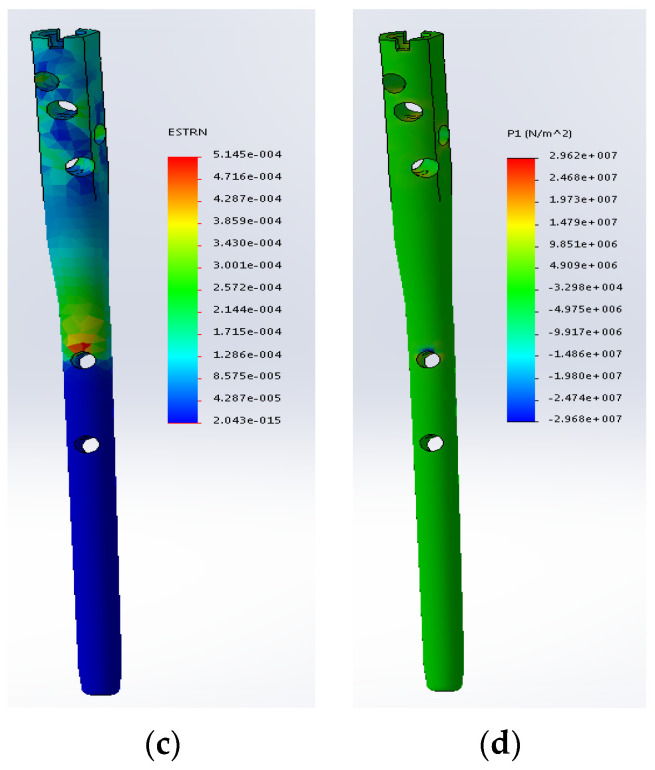
Results of the static finite element analysis performed on the intramedullary nail. (**a**) Von Mises equivalent stresses; (**b**) Displacements; (**c**) Equivalent von Mises strains; (**d**) Principal stresses along the Ox direction.

The finite element analysis provided key insights into the stress and strain distribution within the nail. The simulation results (Figure 20), suggest an absence of plastic deformation during loading, which correlates with the observations derived from the experimental curve (Figure 18).

The displacements (Figure 20b and the strains in Figure 20c,d) indicate that the nail does not reach the threshold for plastic deformation, with stress values remaining well below the material’s yield strength.

**Figure 21:**
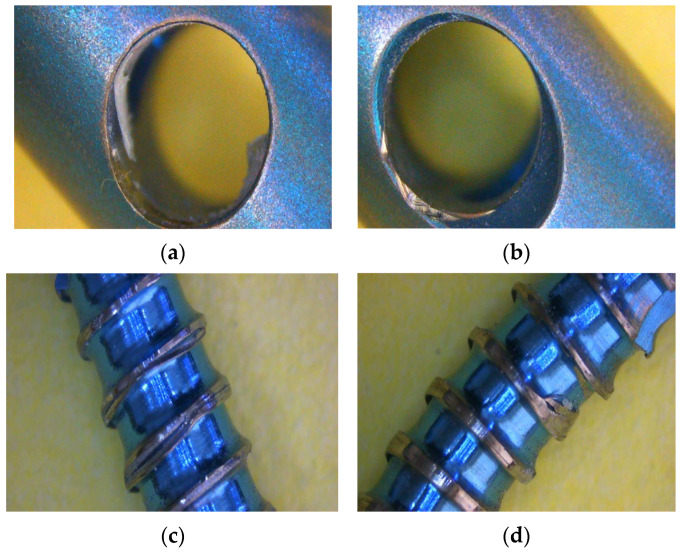
Detailed views of various components within assembly A3. (**a**,**b**) Plastic deformations of diaphyseal fixation holes; (**c**,**d**) Plastic deformations of screws.

The stress distribution on the nail allows for an assessment of how loads are distributed throughout the entire assembly: the load is transferred via the fixation screws from the proximal humerus to the nail, subsequently concentrating in the area of the diaphyseal fixation hole. This stress concentration is further transferred to the screw. Correlating these observations with the macroscopic evidence, the highly stressed regions around the holes and the most severely affected components—the screws—become apparent (Figure 21).

Clear plastic deformations (Figure 21a,b) and thread damage (Figure 21c,d) are visible on the screws. The occurrence of these deformations is considered to have taken place following the failure of the artificial bone.

**Figure 22:**
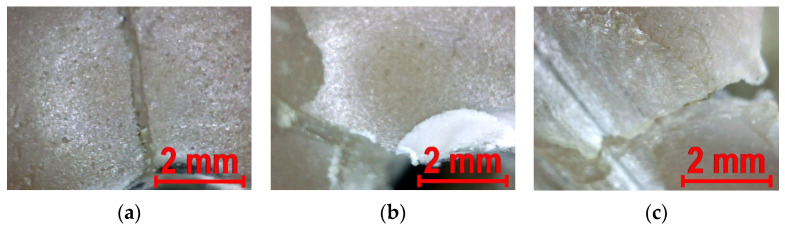
(**a**–**c**) Detailed views of failure location in assembly A3.

For assembly A3, the force–displacement curve indicated a structural failure of one of the components. Macroscopic analysis identified the failure within the artificial bone, with the fracture being clearly visible (Figure 22).

**Figure 23:**
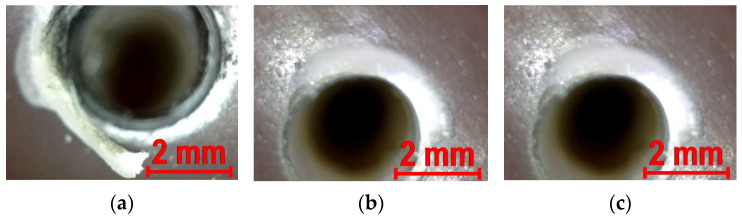
(**a**–**c**) Plastic deformations in the reamed regions.

In addition to the evident fracture indicating bone failure, the reamed regions of the bone exhibit distinct signs of plastic deformation and failure, further supporting the assumption of its structural collapse (Figure 23).

In the case of assembly A3, a stress concentration is observed in the region of the fixation screw located in the medial portion of the bone. As the stress values exceed the strength characteristics of the material used for the artificial bone, structural failure occurs.

### 3.4. Comparative Analysis of the Experimental Results

**Figure 24:**
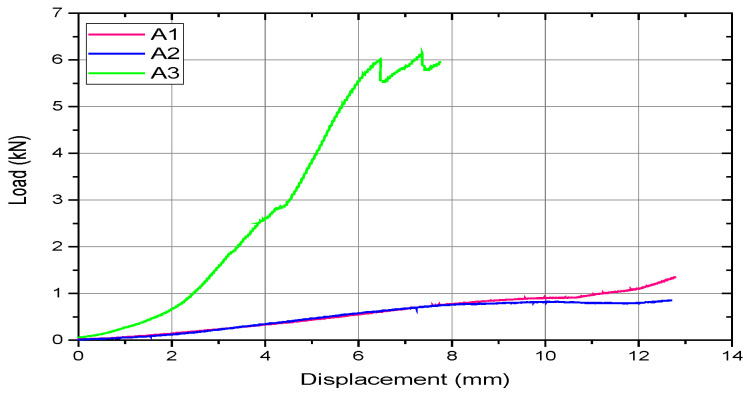
Comparative force–displacement curves obtained from the mechanical testing of the three studied assemblies.

A comparative analysis of the results is conducted based on the mechanical behavior of the assemblies and the strength parameters determined by processing the experimental data. Figure 24 graphically represents the force–displacement curves obtained from the mechanical testing of the three studied assemblies.

In terms of mechanical behavior, assemblies A1 and A2 exhibit similar responses with comparable values, indicating the plastic deformation of a plate component, as previously demonstrated. Conversely, assembly A3, utilizing intramedullary nail fixation, displays marked stiffness and a brittle failure mode, with an absence of plastic deformation.

**Figure 25:**
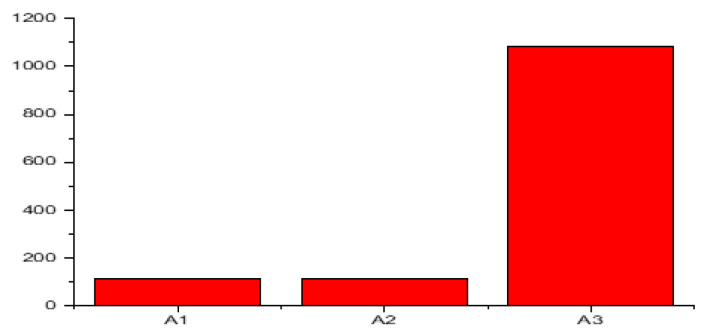
Stiffness variation across assemblies A1, A2, and A3 (N/mm).

Regarding structural stiffness, assemblies A1 and A2 show similar values, whereas the stiffness of assembly A3 is approximately 9.5 times higher (Figure 25).

**Figure 26:**
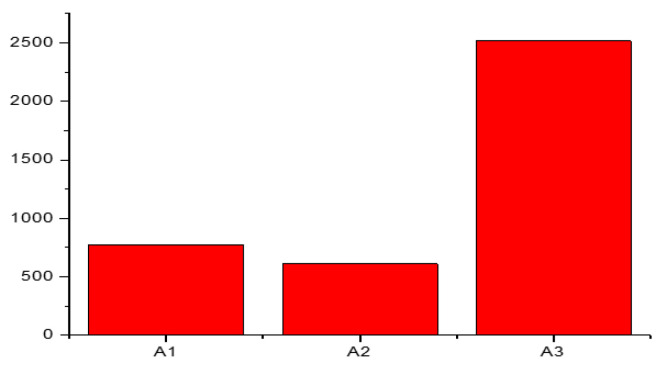
Proportionality limit between force and displacement across assemblies A1, A2 and A3 (N).

Regarding the proportionality limit between force and displacement, a trend similar to that of stiffness is observed: assemblies A1 and A2 show comparable values, with A1 being approximately 163 N higher. The highest value is recorded for assembly A3, which is approximately 4.1 times greater than that of assembly A2 and 3.25 times greater than that of assembly A1 (Figure 26).

## 4. Discussion

The management of proximal humerus fractures remains a controversial subject, particularly in elderly patients with osteoporotic bone, in whom the stability of internal fixation is difficult to estimate in vivo [15,16,17]. As the most mobile ball-and-socket joint, the shoulder presents major challenges in replicating physiological conditions in vitro [18,19]. Our study comparatively evaluated three types of configurations (A1, A2, A3) for fixing a complex Neer 4 fracture, employing a hybrid approach: axial mechanical testing, finite element analysis (FEA), and stereomicroscopy (macroscopic analysis). This complex methodology allowed not only for stiffness evaluation but also for identifying critical stress transmission zones, surpassing the limitations of simple mechanical testing [20,21,22]. However, the findings of the present study should be interpreted within the context of its exploratory experimental design and limited sample size. Given the destructive testing protocol and the limited number of experimental constructs evaluated for each fixation configuration, the obtained results are intended primarily as preliminary biomechanical observations regarding construct behavior and stress-transfer mechanisms under the tested experimental conditions.

Beyond construct-level mechanics, the clinical value of any fixation strategy also depends on the quality of interdisciplinary communication and on how treatment performance is monitored during follow-up, particularly in increasingly digitalized healthcare environments [23,24].

Over the past two decades, locking plates have been considered the gold standard, offering superior biomechanical performance compared to conventional plates [25,26]. However, our results highlight a clear distinction between extramedullary (plates) and intramedullary (nails) systems. While assemblies A1 and A2 (plates) exhibited similar behaviors characterized by plastic deformation of the implant, assembly A3 (intramedullary nail) demonstrated a stiffness approximately 9.5 times higher. This observation correlates with studies by Strasser et al. [27] and Boyer et al. [28], which suggest that the intramedullary nail may represent a potentially advantageous alternative in unstable fractures, particularly with regard to medial support and resistance to varus displacement.

A central issue in biomechanics is finding the balance between stiffness and flexibility. While overly rigid implants may pose a high risk of stress shielding [29], in the case of osteoporotic bone, high primary stability is critical to prevent fixation failure [30]. In the present study, assembly A3 exhibited a brittle failure mode at the bone level without nail deformation, whereas for the plates, failure was induced by the plastic deformation of the implant. This increased resistance of the nail to axial loads is due to the load-sharing principle, as the nail is located closer to the bone’s neutral axis, transferring forces through locking screws to the diaphysis [31,32]. Macroscopic analysis confirmed that in the case of A3, stress concentration around the fixation holes exceeds the strength of the artificial bone before the nail reaches its yield point.

Utilizing finite element analysis in parallel with destructive testing allowed for the qualitative correlation of the critical zones identified experimentally. Our results support the hypothesis that primary stability is decisively influenced by medial support and screw configuration [33,34]. Although locking plates remain a versatile option [35], the incidence of complications such as intra-articular screw penetration [36,37] justifies the exploration of intramedullary systems or cement augmentation in cases of poor bone quality [38,39,40]. The stiffness values obtained in this in vitro study may provide useful biomechanical reference points for understanding construct stability and implant-bone load transfer during the early postoperative period [41,42].

A critical component in assembly stability is the interaction between the screws and the fixation plate. In our study, we observed that the failure of plate assemblies (A1 and A2) was preceded by plastic deformation of the screws and thread damage, confirming the hypothesis that locking screw stability reduces frictional forces compared to conventional screws [43]. However, as indicated in the literature [32], in locking plates, failure is often determined by the simultaneous pull-out of the entire screw construct, an aspect we observed macroscopically in highly stressed areas. This increased pull-out resistance is an advantage under moderate loads but can lead to “disastrous” consequences under high loading forces [36,37], a behavior clearly reflected in the performance gap between the studied plates and the A3 intramedullary nail.

The absence of medial support is frequently associated with varus collapse and intra-articular screw penetration [33]. Under the tested experimental conditions, assembly A3 demonstrated increased construct stiffness and resistance to deformation, suggesting that the intramedullary nail configuration may provide improved intrinsic medial support compared to the evaluated plate constructs. However, this observation should be interpreted cautiously, as the present study did not include osteoporotic bone models or direct comparison with augmentation techniques using grafts or cortical struts reported in other studies [33]. From a translational perspective, future implant-oriented research may also benefit from multifunctional biomaterial concepts aimed at improving the local biological environment and potentially reducing implant-related complications [44]. However, there is an ongoing debate regarding “ideal stiffness” [41,45]. While some authors recommend semi-rigid implants to avoid high stress on osteoporotic bone [29,30], the stiffness values obtained for A3 (1084 N/mm) suggest increased primary stability under the tested axial loading conditions that could facilitate early mobilization—a key objective in treating elderly patients [30,46].

The correlation between in vitro biomechanical tests and FEA simulations allowed for the identification of high-stress regions that are not always visible through external inspection. A qualitative correlation was observed between the numerical simulations and the experimentally identified deformation and failure regions. The stress concentration zones predicted by the finite element analysis corresponded to the areas where macroscopic deformation and experimentally induced structural failure were observed during mechanical testing. Using simplified models for each configuration supported the identification of stress concentrations around the diaphyseal fixation holes for the nail and at the surgical neck for the plates [20,21,22,47]. This multidisciplinary approach confirms that elastic stiffness is a viable parameter for measuring early stability, while destructive testing to failure remains a priority for evaluating secondary stability and the risk of clinical revision [36,48,49].

Several limitations of the present study should be acknowledged. First, the experimental design was based on a limited number of tested constructs, which restricts the possibility of statistical analysis and limits the interpretation of the findings to preliminary biomechanical observations. Second, the mechanical testing protocol employed only axial compression loading and did not reproduce the complex multidirectional loading conditions encountered in vivo at the shoulder joint, including torsional, cyclic, and muscle-driven forces. Third, the finite element analysis was intentionally simplified and focused primarily on the implant components without including a complete bone–implant–screw construct or contact interactions between implant components and bone structures. Consequently, the numerical simulations should be interpreted primarily as complementary qualitative analyses rather than fully predictive computational models. Finally, no osteoporotic bone models or cadaveric specimens with quantified bone mineral density were included in the present study, limiting the direct clinical extrapolation of the obtained biomechanical findings.

## 5. Conclusions

This study provides a comparative biomechanical evaluation of intra- and extramedullary fixation systems for complex Neer 4 proximal humerus fractures, utilizing a hybrid methodology that integrated experimental testing with numerical analysis and stereomicroscopy. In the locking plate configurations, both monoaxial and polyaxial variants exhibited substantially similar mechanical behavior, primarily characterized by bending stress and reaching the plastic deformation threshold at loads of approximately 775 N and 610 N for the polyaxial and monoaxial configurations, respectively. This suggests that, in this single-specimen study, the geometric modifications of polyaxial plates did not significantly alter the load transfer mechanism.

In contrast, intramedullary nail fixation demonstrated a stiffness approximately 9.5 times higher than that of the locking plate systems, maintaining a predominantly axial compression regime. In this configuration, the experimentally observed failure mechanism appeared to involve the bone–screw interface, where stress concentrations may exceed the material strength of the composite bone model.

From a clinical perspective, the obtained findings suggest that fixation behavior may be influenced by bone quality. While the intramedullary nail configuration demonstrated increased construct stiffness under the tested experimental conditions, locking plates may provide a more favorable load-transfer behavior in osteoporotic bone conditions by transferring a greater proportion of the mechanical load to the implant. However, this potential clinical implication should be interpreted with caution, as no osteoporotic bone models or cadaveric specimens with quantified bone mineral density were included in the present study.

The findings of the present study should be interpreted within the limitations of the simplified experimental and numerical design. Further investigations using larger experimental series, multidirectional loading protocols, and more detailed bone–implant computational models are necessary to validate the clinical relevance of these biomechanical observations.

Future studies should try to improve these points. A larger number of specimens should be used in each fixation group so that proper statistical comparisons can be made. It would also be useful to include cadaveric bones with measured bone mineral density, in order to better reflect fixation performance in osteoporotic bone. Cyclic fatigue testing, with repeated loading over many cycles, would provide important information about secondary stability and the long-term risk of hardware failure or loss of reduction, which cannot be captured by a single destructive test. In addition, the experimental setup would become more clinically relevant if it included other loading types, such as torsion, varus bending, and combined loading conditions related to shoulder abduction and rotation. From a computational point of view, future finite element models should include more realistic bone geometry, ideally based on CT data or bone density mapping, so that the full bone-implant-screw system can be analyzed and stress concentrations at the bone-screw interface can be predicted more accurately. Future clinical comparative studies may also benefit from digital and multimodal monitoring tools, which could improve longitudinal follow-up and allow a more consistent capture of patient-related outcomes after fixation. More refined quantitative outcome descriptors may also strengthen future translational studies, as strain-based assessment has become increasingly important across other areas of medical research when subtle functional differences need to be captured objectively. Finally, these biomechanical findings should be followed by prospective clinical comparative studies that assess functional results, complication rates, and revision surgery in similar patient groups treated with polyaxial locking plates or intramedullary nails for Neer IV proximal humerus fractures, especially in osteoporotic bone.

## Data Availability

The experimental data and finite element analysis results supporting the findings of this study are available from the corresponding author upon reasonable request.
